# Brain structure in children with congenital visual disorders and visual impairment

**DOI:** 10.1111/dmcn.14322

**Published:** 2019-08-08

**Authors:** Joe Bathelt, Naomi J Dale, Michelle de Haan, Chris A Clark

**Affiliations:** ^1^ Department of Psychology University of Amsterdam Amsterdam the Netherlands; ^2^ UCL Great Ormond Street Hospital Institute of Child Health University College London London UK; ^3^ Great Ormond Street Hospital for Children NHS Foundation Trust London UK

## Abstract

**Aim:**

To examine if congenital visual impairment is associated with differences in brain anatomy in children.

**Method:**

Ten children (8–12y) with congenital disorders of the peripheral visual system with severe visual impairment (SVI; >0.8 logMAR) or mild‐to‐moderate visual impairment (MVI; 0.6–0.8 logMAR) were compared to 21 typically sighted comparison (TSC) children. Thalamus volume, grey matter density, white matter microstructure, and integrity of visual tracts were investigated in SVI, MVI, and TSC groups with anatomical and diffusion‐weighted magnetic resonance imaging.

**Results:**

Compared to the TSC group, the SVI group had lower white matter integrity in tracts of the visual system (optic radiations: SVI 0.35±0.015, TSC 0.39±0.007 [*p*=0.022]; posterior corpus callosum: SVI 0.37±0.019; TSC 0.42±0.009 [*p*=0.033]) and lower left thalamus volume (SVI 4.37±0.087; TSC 4.99±0.339 [*p*=0.015]). Neuroanatomical differences were greater in the SVI group, while no consistent differences between the MVI and TSC group were observed.

**Interpretation:**

Posterior tracts of the visual system are compromised in children with congenital visual impairment versus those who are typically sighted. The severity of visual input appears to have affected neuroanatomical development as significant reductions were only found in the SVI group.

**What this paper adds:**

- Severe visual impairment in mid‐childhood is associated with reduced integrity of visual pathways and reduced thalamus volume.

## What this paper adds

- Severe visual impairment in mid‐childhood is associated with reduced integrity of visual pathways and reduced thalamus volume.

This article is commented on by Bauer on page https://doi.org/10.1111/dmcn.14339 of this issue.

**Abbreviations**
CDPVS: Congenital disorders of the peripheral visual system
FSL: FMRIB Software Library
MVI: Mild‐to‐moderate visual impairment
PCC: Posterior corpus callosum
ROI: Region of interest
SVI: Severe visual impairment
TSC: Typically sighted comparison

**What this paper adds**

- Severe visual impairment in mid‐childhood is associated with reduced integrity of visual pathways and reduced thalamus volume.

A large proportion of the cortex is dedicated to the processing of visual information in typically sighted children and adults. Visual experience in development appears vital for establishing the necessary architecture in visual cortical areas that support visual processing throughout life.^1^ In cases where children are born with disorders that affect the sensory part of the visual system, the majority are medically untreatable with immediate and chronic absent or reduced vision. While the central nervous system (CNS) is not anticipated to be directly affected in the aetiology of many congenital disorders affecting the peripheral visual or ocular system,^2^ brain structure and function may adjust or be compromised after the visual deprivation caused by the deficits in the sensory parts. The current study therefore aimed to investigate if there are differences in brain anatomy in school‐aged children born with disorders of the peripheral visual system. It was hypothesized that severity of visual impairment may also have an influence on brain development.

Congenital visual impairment has been found to be associated with several structural and functional brain differences in adulthood. The strongest effects have been found in neural structures that are involved in visual processing. The optic nerve and optic chiasm are reduced in volume, as well as alterations in microstructural organization of their white matter.^3^ Thalamic nuclei that are typically involved in the transmission of visual information show reorganization in animal models,^4^ and appear smaller on structural magnetic resonance imaging (MRI) of adults with anophthalmia.^5^ The optic radiations that transfer visual information between the thalamus and the primary visual cortex are reduced in size and show reductions in microstructural integrity.^6^, ^7^ The primary visual cortex also shows a different morphology of reduced grey matter content, reduced cortical surface area, and increased cortical thickness.^3^, ^8^ Even cortical areas that are associated with higher‐order visual processing have shown anatomical differences.^6^, ^9^ Most of this brain research focused on those who are defined as ‘blind’, which encompasses those who are Braille users with estimated visual acuity of 3/60 (logMAR 1.3) or worse,^10^ and did not examine the outcomes of differing levels of visual impairment, including mild‐to‐moderate ranges.

We investigated the relationship of congenital visual impairment with structural brain organization during middle childhood and, specifically, compared the brain organization associated with differing levels of visual impairment. The study focused on children aged 8 to 12 years who had congenital disorders of the peripheral visual system (CDPVS), i.e. disorders of the globe, retina, or anterior optic nerve, and vision level of severe visual impairment (SVI) or mild‐to‐moderate visual impairment (MVI). According to the existing adult literature, we predicted differences in brain structures, particularly in regions associated with vision processing, in children with visual impairment versus those with typical sight and greater differences in those with SVI versus MVI. In addition to whole‐brain comparisons, we focused on the thalamus, optic radiations, and corpus callosum, as highlighted in the adult literature.

## Method

### Participants

The analysis presented here were part of a wider cross‐sectional study on the neural and cognitive sequelae of congenital visual impairment during mid‐childhood. This study was performed in accordance with the Declaration of Helsinki. The study was approved by the National Health Service Research Ethics Committee (12/LO/0939). All parents provided written informed consent for participation and publication of results, and children provided verbal assent for participation.

The inclusion criteria were as follows: (1) children with CDPVS and no other known CNS disorder in the paediatric diagnosis, according to ophthalmological report; (2) age 8 to 12 years; (3) congenital visual impairment, with estimated best‐corrected visual acuity of logMAR 0.6 or worse; (4) verbal IQ greater than 75 or attending mainstream school and performing at age‐appropriate level; and (5) English as their first language. Exclusion criteria were preterm birth (<37wks’ gestational age), cerebral visual impairment, ‘complex’ CDPVS (including known CNS involvement), endocrine abnormalities, epilepsy, and additional neurological impairments, e.g. motor.

Recruitment was undertaken through initial identification via the patient database of the tertiary developmental vision neurodisability clinic (Great Ormond Street Hospital for Children), which is the primary research site, or the local collaborator of a tertiary children's eye hospital (Moorfields Eye Hospital) and an open recruitment call through charitable and educational agencies associated with visual impairment. Parents could also self‐refer. Information on the child's ophthalmological disorder and vision level were obtained from medical notes by the clinician (ND or collaborator).

Children in the typically sighted comparison (TSC) group were recruited via local advertisement for a parallel study that used the same MRI protocol. Inclusion criteria included same age and similar level of verbal intelligence, normal or corrected‐to‐normal vision, and English as a first language. Children were excluded if they had any known neurological or psychiatric conditions.

Congenital childhood vision disorders with SVI are rare, with an estimated prevalence of less than 2 to 3 per 10 000 live births in the UK, rising to 5 to 6 during childhood.^11^ The current study focuses on children with CDPVS and no other central brain involvement according to their paediatric report (referred to as ‘potentially simple’ CDPVS),^2^ which is estimated to form about half of congenital vision disorders in the first year of life. A smaller proportion of these children may also have intellectual disability,^2^ so identification of patients who are in the ‘normal’ intellectual range raises significant challenges for recruitment.

In the wider study, 18 children with visual impairment consented (via parents) to participate over the duration of the study (2012–2014). Twelve of the children with visual impairment participated in this part of the study. The remaining six children did not participate because their parents were concerned about safety or time commitment. Of those participating, two (one with MVI, one with SVI) had to be excluded from final analysis owing to poor data quality. The final sample consisted of five children with SVI (two females, mean age 10y 5mo [SD 10mo], range 10–12y; see Table 1), five with MVI (two females, mean age 10y 4mo [SD 1y 7mo], range 8–12y), and 21 TSC children (11 females, mean age 10y 10mo [SD 2y], range 8–13y). There was no significant difference in age between the groups (one‐way analysis of variance: F[2,29]=0.23, *p*=0.796). No differences in movement (maximum framewise displacement) were found between the vision level groups (mean SVI 0.84 [standard error {SE} 0.05]; mean MVI 0.8 [SE 0.1]; mean TSC 0.81 [SE 0.09]; *F*_2,28_=0.02, *p*=0.981).

**Table 1:** Characteristics of children with visual impairment

Case	Sex	Age (y)	logMAR	Near detection	Visual fisorder
MVI1	Male	12	0.6		Rod–cone dystrophy
MVI2	Female	8	0.6		Oculocutaneous albinism
MVI3	Male	12	0.6		Congenital nystagmus
MVI4	Male	10	0.7		Ocular albinism, congenital nystagmus
MVI5	Female	12	Left: 0.23, right: light perception only		Unilateral optic nerve hypoplasia
SVI1	Male	12	0.9		Oculocutaneous albinism
SVI2	Male	10	1.2		Leber congenital amaurosis
SVI3	Male	10	1.225		Norrie disease
SVI4	Female	11	–	1.5cm ‘lure’ from 20cm	Leber congenital amaurosis
SVI5	Female	10	–	12.5cm ‘lure’ from 50cm	Bilateral microphthalmia

MVI, mild‐to‐moderate visual impairment; SVI, severe visual impairment.

### Measures

#### Vision level

The experimenter (JB) was trained by a paediatrician specialized in visual impairment to assess visual acuity using the Sonksen logMAR test.^12^, ^13^ Distance acuity at 3m was measured with both eyes open and with corrective glasses if the child regularly wore glasses. Two children with SVI, defined here as vision level of logMAR greater than 0.8, could not identify the largest optotypes on the Sonksen logMAR test (>1.65 logMAR) and were therefore assessed on the Near Detection Scale (cases SVI4 and SVI5, see Table 1).^14^ MVI was defined here as vision level of 0.6 to 0.8 logMAR.

#### MRI data acquisition

All scans were performed on a Siemens Avanto 1.5 T clinical system (Siemens Healthcare, Erlangen, Germany), using a self‐shielding gradient set with maximum gradient strength of 40mT/m and a 32‐channel quadrature head coil. T1‐weighted volume scans were acquired using a whole‐brain coverage three‐dimensional fast low angle shot structural image acquired at 1mm^3^ image resolution (echo time: 4.9ms; repetition time: 11ms). Diffusion MRI was acquired using echo‐planar diffusion‐weighted images with an isotropic set of 60 non‐collinear directions at *b*=1000s/mm^2^, interleaved with four b0 volumes. Whole‐brain coverage was obtained with 60 contiguous axial slices at 2.5mm^3^ resolution (echo time: 89ms; repetition time: 7300ms).

#### Voxel‐based morphometry

Whole‐brain voxelwise comparison of grey matter compartments were performed. Local differences in grey matter volume were analysed with the voxel‐based morphometry pipeline part of the FMRIB Software Library (FSL), an optimized VBM protocol carried out with FSL tools.^15^ The modulated grey matter images were smoothed with an isotropic Gaussian kernel at sigma 3mm. Similar results were obtained at 2mm and 4mm. Group comparisons were carried out using permutation *t*‐tests with 10 000 permutations and cluster‐free threshold enhancement to correct for multiple comparisons using FSL randomise.

#### Thalamus volume

To obtain the volume of the thalamus, a model‐based segmentation and registration approach was used (FSL FIRST). Accurate segmentation of the thalamus was visually inspected for all participants. The total brain volume for each participant was obtained using FSL SIENA.^15^ The relative thalamus volume was calculated by dividing the thalamus volume by the total brain volume.

#### Processing of diffusion MRI data

Diffusion‐weighted imaging allows the quantification of water diffusion in tissue in vivo. Correction for eddy current induced artefacts, motion, and field inhomogeneities were applied using the FSL eddy. Susceptibility artefacts were corrected with FSL topup using the b0 images. The images were then submitted to a non‐local means denoising algorithm in the Diffusion Imaging in Python (DiPy) v0.8.0 package to boost the signal‐to‐noise ratio. Next, a brain mask of the b0 image was created using FSL BET. White matter integrity was quantified as fractional anisotropy.

#### Tract‐based spatial statistics

Differences in white matter integrity were compared between the groups using tract‐based spatial statistics.^16^ Group comparisons were carried out using FSL randomise with 10 000 permutations and cluster‐free threshold enhancement.

#### Tractography of the optic radiation and posterior corpus callosum

**Figure 1:**
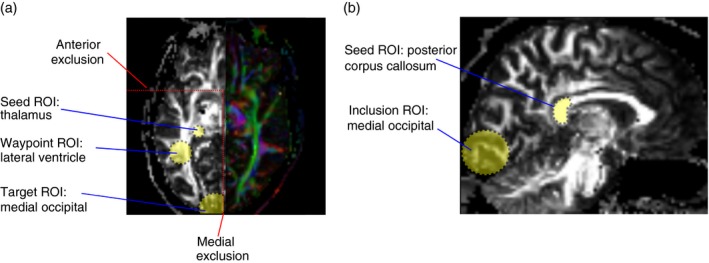
Illustration of region of interest (ROI) used for tractography of (a) the optic radiations and (b) the posterior corpus callosum. [Colour figure can be viewed at http://www.wileyonlinelibrary.com]

For reconstruction of the optic radiation, seed and inclusion regions were defined on fractional anisotropy maps for each participant in subject space.^17^, ^18^ Specifically, a spherical seed region of interest (ROI) with a 3mm radius was defined in the white matter adjacent to the thalamus (see Fig. 1). A second ROI with a 6mm radius was placed in the white matter adjacent to the lateral ventricles a few slices dorsally from the seed ROI. Another inclusion ROI with 20mm radius was placed in the occipital lobe centred near the calcarine fissure. Exclusion ROIs were placed at the level of the anterior tip of the brain stem in axial view, ventrally to exclude streamlines of the corticospinal tract, and along the midline to avoid fibres of the corpus callosum. In addition, a brain mask was used for exclusion.

For reconstruction of the posterior corpus callosum (PCC), a seed mask was defined on the posterior quarter of the corpus callosum on a sagittal slice. To define the posterior quarter, the number of voxels greater than 0.1 fractional anisotropy were counted on a medial slice and divided into equal‐length parts (see Fig. 1). This segment of the corpus callosum has been found to mostly contain fibres from visual areas in both hemispheres. In addition, an inclusion ROI consisting of a 20mm sphere placed over the medial occipital cortex was defined in each hemisphere.

Probabilistic tracking was performed based on a constrained spherical deconvolution model in MRTrix,^19^ with a target of 1000 streamlines, step size of 0.2mm, maximum curvature of 1mm and minimum/maximum length of 10mm/200mm. Streamlines were set to terminate in voxels with fractional anisotropy <0.1 or when hitting an exclusion mask. After probabilistic tracking, a visitation map of all voxels that contained at least 100 streamlines was calculated. This cut‐off produced a satisfactory reconstruction of the optic radiations and PCC in all participants.

### Statistical analysis

Because vision was assessed with different assessments, owing to children with very low vision not seeing the logMAR optotypes, that do not translate into a single continuous measure of acuity, statistical models used group comparisons between children with SVI, MVI, or typical sight rather than using visual acuity as a continuous variable. Statistical comparisons of ROI values were based on the Kruskal–Wallis test with a factor for group (SVI, MVI, TSC) followed by a post hoc Dunn test for group contrasts carried out using the scikit‐posthocs package v0.6.1 for Python. A significance threshold of *p*_corrected_<0.05 was set for all analyses. Bonferroni correction was used to account for multiple comparisons.

## Results

### Participant characteristics

Table 1 shows characteristics of degree of visual impairment, visual disorder, etc. In the MVI group, one child had better vision than the MVI range in one eye (MVI5). In the SVI group two children were in the ‘blind’ range (vision of 1.3 logMAR or worse: SVI4 and SVI5), but they had some minimal detection vision.

Anatomical MRI scans were obtained from the 10 children with visual impairment; visual inspection of the images indicated no gross structural abnormalities in eight of the 10 children. Reduction of the right optic nerve was apparent in one child; small eyes were apparent in another child (see Table 1 for diagnostic information).

### Whole‐brain comparison of grey matter volume

There was no statistically significant difference in total grey matter volume between the vision groups (SVI: mean 904.26cm^3^ [SE 38.841cm^3^]; MVI: mean 982.45cm^3^ [SE 41.625cm^3^]; TSC: mean 1000.44cm^3^ [SE 10.952cm^3^]; MVI vs TSC: *p*=0.454; SVI vs MVI: *p*=0.745). There was a trend‐level difference in total grey matter with lower values in the SVI group than in the TSC group (SVI vs TSC: *p*=0.091). Voxelwise comparison indicated no significant between‐group differences after correction for multiple comparisons. The corrected statistical map showed a cluster of decreased grey matter in the SVI group versus TSC at *p*_corrected_=0.08. This cluster was adjacent to the optic radiations and seemed to extend into the white matter, indicating that this may be an artefact of white matter differences.

### Comparison of thalamus volume

The volume of the thalamus was compared between the vision groups. Statistical comparison indicated a trend‐level difference between the groups for the relative volume of the left thalamus (Kruskal–Wallis test: *H*=5.29, *p*=0.071). Follow‐up contrasts indicated a smaller left thalamus volume, corrected for whole‐brain volume, in the SVI group versus the TSC group (*p*_corrected_=0.047, see Table 2). No differences were indicated for the right thalamus (Kruskal–Wallis test: *H*=2.81, *p*=0.245).

**Table 2:** Comparison of thalamus volume

	Left thalamus				Right thalamus			
	Absolute volume (mm^3^)		Relative volume (1×10^4^)		Absolute volume		Relative volume	
	Mean	SE	Mean	SE	Mean	SE	Mean	SE
SVI	7944.95	147.564	4.37	0.087	7810.29	1143.179	4.35	0.087
MVI	7830.80	605.227	4.67	0.309	7603.60	635.935	4.67	0.330
TSC	9145.60	583.092	4.99	0.339	8713.80	503.056	4.97	0.341

Comparison				*p*				*p*
SVI vs TSC				**0.047**				0.074
MVI vs TSC				0.871				0.626
SVI vs MVI				0.175				0.175

The statistical comparison was based on relative volume corrected for brain volume. Bonferroni–corrected *p*‐values are reported. Bold type indicates significance. SE, standard error; SVI, severe visual impairment; MVI, mild‐to‐moderate visual impairment; TSC, typically sighted comparison.

### Whole‐brain comparison of white matter microstructure

**Figure 2:**
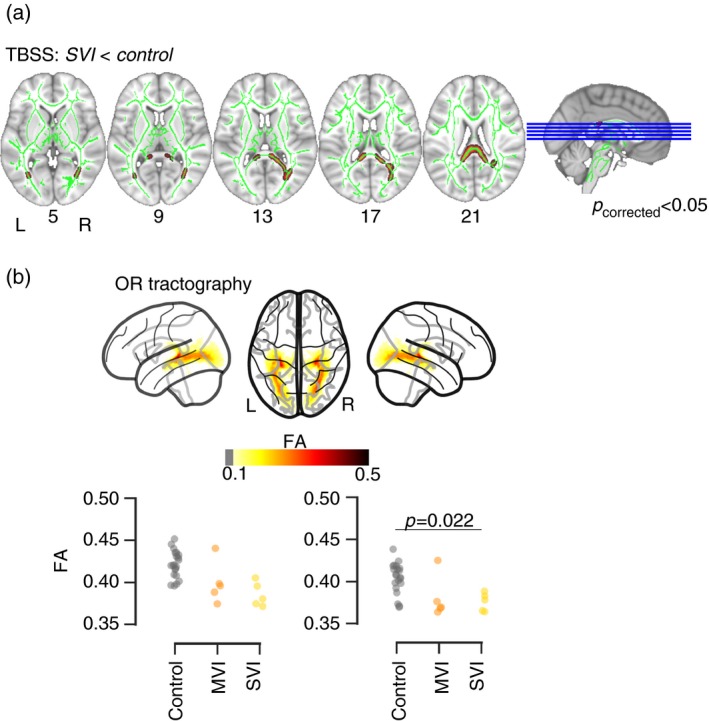
Results of group‐wise comparisons. (a) Comparison of fractional anisotropy (FA) with tract‐based spatial statistics (TBSS). The green lines indicate the white matter skeleton. The red overlay shows voxel with significantly lower FA in the severe visual impairment (SVI) group compared with typically sighted controls (TSC) in the optic radiations (OR) and posterior corpus callosum. (b) Comparison of FA within the OR reconstructed using tractography within individual participants. The top panel illustrates the reconstruction of the OR projected into Montreal Neurological Institute space. The scatter plot shows the values of FA within the left and right OR in the SVI, mild‐to‐moderate visual impairment (MVI), and TSC group. [Colour figure can be viewed at http://www.wileyonlinelibrary.com]

There was no significant difference between the groups in total white matter volume (SVI: mean 5911mm^3^ [SE 482mm^3^]; MVI: mean 5525mm^3^ [SE 113mm^3^]; TSC: mean 5983mm^3^ [SE 184mm^3^] [*H*=3.66, *p*=0.159]). Differences in white matter microstructural organization across the whole brain were investigated. Tract‐based spatial statistics analysis indicated a significant reduction of fractional anisotropy around the optic radiations and the PCC in the SVI group compared with TSC (see Fig. 2; statistics using cluster‐free threshold enhancement – left optic radiation: *p*_corrected_=0.048; right optic radiation: *p*_corrected_=0.006, PCC: *p*_corrected_=0.038). There were no significant differences after correction for multiple comparisons for the MVI versus TSC, or MVI versus SVI comparisons.

### Tractography of the optic radiations and PCC

Statistical analysis of fractional anisotropy within the reconstructed optic radiations indicated significant differences between the groups (Kruskal–Wallis test, left optic radiation: *H*=9.38 [*p*=0.009]; right optic radiation *H*=11.81 [*p*=0.003]). Follow‐up contrasts indicated lower fractional anisotropy in the SVI than in the TSC group in the left and right optic radiations (see Table 3). When excluding one apparent extreme data point in the MVI group, there was a significant difference between the MVI and TSC group in the left and right optic radiations (left: mean 0.39 [SE 0.005; *p*=0.003]; right: mean 0.37 [SE 0.003; *p*=0.006]).

**Table 3:** Comparison of fractional anisotropy within the optic radiations

	Left optic radiation		Right optic radiation	
	Mean	SE	Mean	SE
SVI	0.38	0.029	0.35	0.015
MVI	0.34	0.030	0.36	0.029
TSC	0.42	0.007	0.40	0.007

Comparison		*p*		*p*
SVI vs TSC		**0.002**		**0.006**
MVI vs TSC		0.055		0.055
SVI vs MVI		0.347		0.754

Bold type indicates significance. SE, standard error; SVI, severe visual impairment; MVI, mild‐to‐moderate visual impairment; TSC, typically sighted comparison.

Comparison of fractional anisotropy within the PCC indicated a significant difference between the groups (Kruskal–Wallis test: *H*=5.98; *p*=0.04). Follow‐up contrasts showed a reduction in the SVI group versus the TSC group and the MVI group (see Table 4). Other comparisons did not reach the significance criterion.

**Table 4:** Comparison of fractional anisotropy within the posterior corpus callosum

	Mean	SE
SVI	0.37	0.019
MVI	0.46	0.038
TSC	0.42	0.009

Comparison	*t*	*p*
SVI vs TSC	−2.75	**0.025**
MVI vs TSC	1.35	0.495
SVI vs MVI	−2.11	**0.047**

Bonferroni‐corrected *p*‐values are reported. Bold type indicates significance. SE, standard error; SVI, severe visual impairment; MVI, mild‐to‐moderate visual impairment; TSC, typically sighted comparison.

## Discussion

This study investigated whether school‐aged children with CDPVS show differences in structural brain organization related to the severity of their visual impairment compared with age‐matched TSC children. The sample comprised a group of children with MVI and a group of children with SVI, including some children in the ‘blind’ range according to World Health Organization definitions. The most pronounced differences were found in white matter microstructural organization of the optic radiations and the PCC in children with SVI. There was also a significant reduction in relative volume of the left thalamus in children with SVI. Children with MVI showed no consistent differences compared with the TSC group. The reduction in thalamus volume in children with SVI is in line with other investigations in specific vision disorders.^5^, ^20^ In contrast to other studies,^21^, ^22^ the current investigation did not find significantly reduced grey matter volume around the occipital cortex in the two visual impairment groups. The white matter differences indicated in the optic radiations and PCC are consistent with the findings in the adult literature with SVI/’blind’ levels of vision.^5^, ^9^ This provides further evidence to support the association of SVI with constraints in the development of the white matter structures of the posterior visual system. This association may be related to prolonged maturation of the white matter visual system after birth and its dependency on visual experience for full maturation.^23^ There may be differences in white matter integrity at least in some children, which may reflect earlier severity of visual impairment in infancy and the first year of life, and which warrants further investigation in a longitudinal sample. Differences in the neuroanatomical organization in MVI would also be expected based on reports in other samples of children at risk of visual impairment, e.g. in the context of preterm birth or hormone deficiency.^24^, ^25^

The strengths of this study are the taxonomic clarity of including only children with CDPVS and no other brain involvement according to their paediatric report, a relatively narrow age range of middle childhood, precise measurement of vision level and comparison between SVI and MVI, and application of well‐validated MRI methods. However, the current study has limitations which may affect generalization of the findings. First, the sample size was limited to very small subgroups for each level of visual impairment, due to the challenge of recruiting children with CDPVS and no other brain involvement or intellectual disability and time constraints of the project. Second, to reach a minimum sample size, a range of disorders were included. The individual disorders are extremely rare and heterogeneous with often little‐understood and complex genetic causes that may influence the brain phenotype. The results obtained with this small sample need to be replicated in larger samples, e.g. through international consortia of rare disorder research. Third, no child had profound visual impairment without vision at all, which may have very specific constraining effects on development and associated brain organization.^26^, ^27^

In conclusion, the current study provides first evidence that SVI is associated with reduced white matter microstructural organization in tracts of the brain's visual system (optic radiations, posterior corpus callosum) and differences in thalamus volume in mid‐childhood. These findings suggest that the organization of central visual structures is influenced by the quantity or quality of sensory visual inputs during development before mid‐childhood.
